# An Improved Method for Prediction of Cancer Prognosis by Network Learning

**DOI:** 10.3390/genes9100478

**Published:** 2018-10-02

**Authors:** Minseon Kim, Ilhwan Oh, Jaegyoon Ahn

**Affiliations:** Department of Computer Science and Engineering, Incheon National University, Incheon 22012, Korea; JHflower@inu.ac.kr (M.K.); oih0404@inu.ac.kr (I.O.)

**Keywords:** cancer prognosis, multi-omics, GANs, deep learning, PageRank

## Abstract

Accurate identification of prognostic biomarkers is an important yet challenging goal in bioinformatics. Many bioinformatics approaches have been proposed for this purpose, but there is still room for improvement. In this paper, we propose a novel machine learning-based method for more accurate identification of prognostic biomarker genes and use them for prediction of cancer prognosis. The proposed method specifies the candidate prognostic gene module by graph learning using the generative adversarial networks (GANs) model, and scores genes using a PageRank algorithm. We applied the proposed method to multiple-omics data that included copy number, gene expression, DNA methylation, and somatic mutation data for five cancer types. The proposed method showed better prediction accuracy than did existing methods. We identified many prognostic genes and their roles in their biological pathways. We also showed that the genes identified from different omics data were complementary, which led to improved accuracy in prediction using multi-omics data.

## 1. Introduction

Accurate identification of prognostic biomarkers is an important goal in bioinformatics, because it can increase the accuracy in predicting the prognosis of cancer patients. This leads to provision of more appropriate therapies and development of better drugs for cancer patients. Many bioinformatics methods have been proposed and applied for various types of omics data, including genome, transcriptome, proteome, and epigenome data, to reveal biomarkers that provide more accurate prediction of cancer prognosis.

In the relatively early stages of those approaches, the aim was generally to identify prognostic biomarkers to predict prognoses of cancer patients, by applying statistical [1,2] or machine learning methods [3,4] to gene expression data. In more recent methods, additional biological network information was used to identify prognostic gene modules. The use of these prognostic gene modules increased the prediction accuracy and deepened our understanding of cancer at the molecular level. For example, Langfelder and Horvath identified a prognostic gene module in a weighted correlation network using a trait-based gene significance measure [5], and Wu & Stein used Markov clustering (MCL) and principal component analysis (PCA) to identify prognostic gene modules [6]. We also developed a method named CPR (Clustering and PageRank) [7], which exploits Google’s PageRank algorithm to identify heterogeneous sets of prognostic genes. 

However, these methods still leave room for improvement. In particular, sufficient amounts of multi-omics data, including genome (for single nucleotide polymorphisms (SNPs) or copy numbers) and epigenome data, as well as gene expression data, have been amassed for exploitation. One of the main difficulties of prognosis prediction is the heterogeneity of cancer [8] resulting from genetic instability [9]. To address this heterogeneity, we developed CPR [7], which showed improved accuracy in the prediction of cancer prognosis through clustering of homogeneous samples but had the limitation of not being applicable for multi-omics data, which can provide even more accurate prediction of cancer prognosis.

Many studies have used multi-omics data to address the heterogeneity of cancer. Ovaska et al. proposed a data integration framework known as Anduril, which integrates results from each form of multi-omics data (such as gene expression data, SNP, and DNA methylation data) [10]. However, Anduril is focused more on collecting and processing data than on analyzing data using machine learning and statistical methods [11]. Bonnet et al. [12] reconstructed a module network using multi-omics data. However, to find co-expressed clusters, input data is limited to gene expression data and only one other, different type of data [11].

In the present study, we propose a novel machine learning method for more accurate prediction of prognosis, which improves CPR to allow handling of multi-omics data. We applied the proposed method to copy number alteration data (CNA), gene expression data (mRNA), DNA methylation data, and somatic mutation data (SNP) for five cancer types: pancreatic adenocarcinoma (PAAD), breast invasive carcinoma (BRCA), kidney renal clear cell carcinoma (KIRC), brain lower grade glioma (LGG), and stomach adenocarcinoma (STAD). The proposed method showed better prediction accuracy than did previously existing methods, including CPR. We were also able to identify many novel prognostic biomarkers and predict their roles in biological pathways.

## 2. Materials and Methods 

### 2.1. Data Preparation

First, we downloaded gene mRNA data, CNV data, DNA methylation data, SNP data, and clinical data for PAAD, BRCA, KIRC, LGG, and STAD from The Cancer Genome Atlas (TCGA) [13] using TCGA assembler [14]. The mRNA data, CNV data, DNA methylation data, and SNP data for each sample were normalized using *z*-scores.

Each sample was labeled as a good or poor prognosis sample if the sample source’s vital status was alive, and if that person lived a longer or shorter time, respectively, than the criteria (Table 1). Table 1 summarizes the data for each cancer type. We also downloaded the functional interaction networks (FIs network), which includes known pathways in human biology, from the Reactome database [15,16].

### 2.2. Method

**Figure 1:**
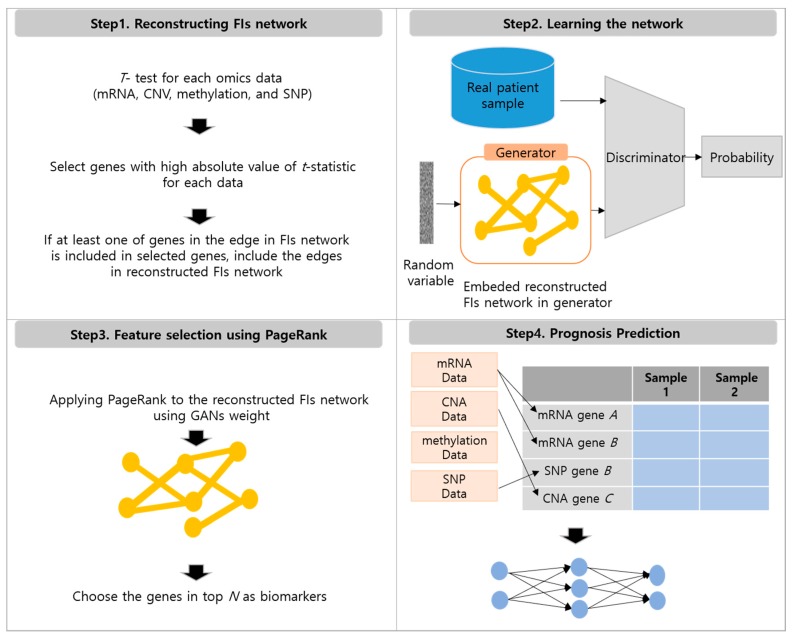
Overview of the method. First, FIs network is reconstructed. Second, reconstructed FIs network is trained using generative adversarial networks (GANs). Third, PageRank algorithm is applied to the reconstructed FIs network using GANs weights, and top *N* biomarkers are selected. Finally, features are selected and patient’s prognosis is predicted using Multi-Layer Perceptron [19].

Our approach consists of four steps, (1) reconstructing FIs network, (2) learning the reconstructed FIs network using generative adversarial networks (GANs) [17], (3) feature selection using PageRank [18] with GANs weights, and (4) prognosis prediction. Each step will be explained in the following sections. Figure 1 summarizes the entire process.

#### 2.2.1. Reconstructing FIs Network 

In each of the mRNA, CNV, DNA methylation, and SNP data sets, we performed a *t*-test comparing good and poor sample groups for each gene. The *t*-test uses the following formula:

(1) $$t_{i}=\frac{\bar{x_{i,p}}-\bar{x_{i,g}}}{s_{i}\cdot \sqrt{\frac{1}{n_{p}}+\frac{1}{n_{q}}}},$$

where $t_{i}$ is the *t*-test statistic of *i*-th gene, $\bar{x_{i,p}}$, $\bar{x_{i,g}}$ are the means of the poor and good sample groups for *i*-th gene, respectively, $n_{p}$, $n_{g}$ are the number of poor and good samples, respectively. Here, $s_{i}$ is calculated using:

(2) $$s_{i}=\frac{\sqrt{\left(n_{p}-1\right)S_{x_{i,p}}^{2}+\left(n_{g}-1\right)S_{x_{i,g}}^{2}}}{n_{p}+n_{g}-2}$$

where $s_{i,p}$ and $s_{i,g}$ are the standard deviations of the poor and good samples for each *i*-th gene, respectively. In each of the four types of data, we took the absolute value of the *t*-test statistic and we selected the top *N* genes that showed statistical differences between the means of good and poor sample groups. Those *N* genes using mRNA, CNV, DNA methylation, and SNP data were denoted as *M*, *C*, *T*, and *S*, respectively. Let *U* be the union of *M*, *C*, *T*, and *S*, and the *i*-th edge of the FIs network as $e_{i}$ and the gene pair comprising $e_{i}$ as $a_{i}$, $b_{i}$, respectively. For all edges of the FIs network, if $a_{i}\in U\;\mathrm{or}\;b_{i}\in U$, then $e_{i}$ is included in the reconstructed FIs network.

#### 2.2.2. Learning the Reconstructed FIs Network Using Generative Adversarial Networks

The GANs model consists of a generator that generates data by learning the distribution of real data and a discriminator that distinguishes between actual data and fake data [17]. The generator receives more training regarding how to generate data that is similar to real data, while the discriminator receives more training regarding how to distinguish between real and fake data. The GANs model is trained through this competition.

To enable learning of the real data distribution in the generator, noise variables $P_{z}\left(Z\right)$ from the standard normal distribution are input to the generator. The generator $G\left(Z;\theta_{g}\right)$ then maps the input data into a new space. *G* is a Deep Neural Network (DNN) with parameters $\theta_{g}$. The discriminator is defined as $D\left(x;\theta_{d}\right)$ and *D* is also a DNN with parameters $\theta_{d}$. The D(x) outputs a probability that data x is real. 

The discriminator and generator are competitively trained to minimize log(1−D(G(z))). The loss function of the discriminator and generator can be expressed as follows:

(3) $$\begin{array}{c} \min\\ G \end{array}\begin{array}{c} \mathrm{maxi}\\ D \end{array}V\left(D,G\right)=\;E_{x\sim p_{data}\left(x\right)}[\log D\left(x\right)]+E_{z\sim p_{z}\left(z\right)}[\log(1-D\left(G\left(z\right)\right))].$$

Let G=(V,E) be a reconstructed FIs network with *N* genes. *V* represents the gene and *E* represents the connected edge between the genes. *G* is a unidirectional graph, and can be expressed in a symmetric adjacent matrix, *A*, which can be expressed as follows:

(4) $$A_{ij}=\{\begin{array}{l} 1,\;\mathrm{if}\;V_{i}\;\mathrm{and}\;V_{j}\;\mathrm{are}\;\mathrm{connected},\;\forall i,j=1,\ldots ,n\\ 0,\;\mathrm{otherwise}. \end{array}$$

Generally, a generator learns the distribution of the real data using a fully connected network, but in the present study, we used a reconstructed FIs network instead of a fully connected network. Our generator includes input and output layers but has no hidden layers. The number of neurons in the input and output layers is the number of genes in the reconstructed FIs network. The matrix that connects the input layer to the output layer is represented by a sparse matrix *A*, which is a reconstructed FIs network.

Our discriminator includes input, hidden, and output layers. The number of neurons in each layer is the number of genes in the reconstructed FIs network, 256, and 1 respectively. Activation functions in the hidden and output layers are ReLu and sigmoid, respectively.

The input of the generator is of *N*-dimensional vectors of noise randomly extracted from the standard normal distribution. The output of the generator is also of *N*-dimensional vectors. The generator in a GANs model can be described as follows:

(5) $$\mathrm{Output}\;=\;\sigma \left(X\left(A⊙w⊙w^{T}\right)\right),$$

where symbol ⊙ denotes a Hadamard (element-wise) product, σ is a ReLu function, *X* is the input vector, and *W* is a randomly initialized edge weight in the network. Because the reconstructed FIs network is symmetric, $A_{ij}=A_{ji}$ must be satisfied; therefore, we create a symmetric matrix through $w⊙w^{T}$. Then, using the reconstructed FIs network as a filter, only the actual edge weights remain. After a training step, the negative edge weights are converted to positive values. As a result, we can learn the distribution of the real data of the cancer patients to compute the weights of the reconstructed FIs network. We provided the hyper parameters used for GANs in Table 2.

We trained GANs using our generator, a fully connected discriminator, and real data created using multi-omics. As real data, we used *N*-dimensional vectors based upon multi-omics data. We created it by selecting one of mRNA, CNV, DNA methylation, and SNP data for each gene with a large absolute *t*-test value. Through Section 2.2.2, we can obtain weights of reconstructed FIs network (generator), which are learned from the multi-omics data.

#### 2.2.3. Feature Selection Using PageRank with Generative Adversarial Network Weights

Next, we applied the PageRank algorithm [18] to the reconstructed FIs network of which weights are learned by the GANs model, to reveal the importance of genes. The importance of genes is determined by their PageRank scores, and each PageRank score is calculated as follows:

(6) $$PR_{i}^{n}=\frac{1-d}{N}+d\sum_{j\in M_{i}}\frac{\left|G_{ij}\right|}{\sum_{t\in M_{j}}\left|G_{tj}\right|}PR_{j}^{n-1},$$

where $PR_{i}^{n}$ is the PageRank score of the *i*-th gene after *N* iterations, *N* is the number of genes, and *d* is a damping factor. $M_{i}$ is the group of genes adjacent to the *i*-th gene, $G_{ij}$ is the absolute value of the weight between the *i*-th gene and the *j*-th gene in the GANs generator. $PR_{i}^{n}$ converges when the following conditions are met:

(7) $$\max\left(\left|PR^{n}-PR^{n-1}\right|\right)<0.005,$$

where max(V) returns the largest element of vector V.

We ranked the genes based on their PageRank scores and selected the top *N* genes, where *N* is a user parameter. To select a stable and robust feature for random initialization of weights, we repeatedly experimented with the reconstructed network learning-phase using GANs and the PageRank process (*t* times). The genes that appeared more than *b* times in *t* experiments were selected as biomarkers (*b* ≤t), where *b* and *t* are also user parameters.

#### 2.2.4. Prognosis Prediction

Next, we determined which dataset had affected each biomarker, using the sets *M*, *C*, *T*, and *S* that we identified in the reconstructing FIs network step. If a gene belonged to only one group among *M*, *C*, *T*, or *S*, then one of mRNA, CNV, DNA methylation, or SNP data for a gene was used as a feature. If a gene belonged to multiple groups among *M*, *C*, *T*, or *S*, then we used all of them as a feature. For example, if gene *A* belonged to *M*, we selected the mRNA data of the gene *A* as a feature. If gene *B* belonged to both *M* and *C*, we used both the mRNA data and the CNV data for gene *B* as features. If a gene did not belong to any group, we counted the number of times that its neighboring genes belonged to *M*, *C*, *T*, or *S*, and used the dataset with the largest count as a feature. For example, if gene *A* did not belong to any group and it had two neighboring genes that belonged to *M* and one neighboring gene that belonged to *C*, then we could use mRNA data for gene *A*. By determining the dataset for each gene, we could create the dataset, as shown in step 4 of Figure 1. After the dataset was constructed, we used Multi-Layer Perceptron [19] for classification, further explained in Section 3.1.

## 3. Results

### 3.1. Parameter Selection

To validate the candidate biomarkers, we performed 10-fold cross-validation. For Multi-Layer Perceptron, we used ReLu as an activation function, and the L2 term for regularization. We summarize all the parameters used for the experiments in Supplementary Table S1. We also provide the selected biomarkers in Supplementary Table S2.

### 3.2. Prognostic Prediction

**Figure 2:**
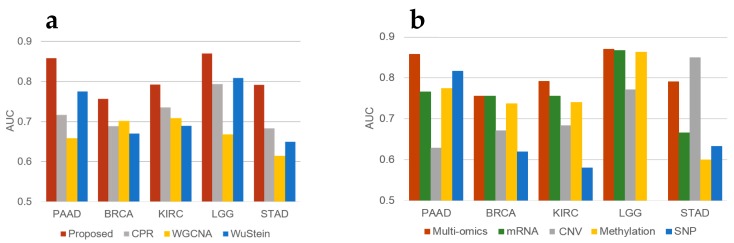
Prognostic accuracy in terms of area under the curve (AUC) for each (**a**) method and (**b**) omics dataset. CPR (Clustering and PageRank), WGCNA (weighted gene co-expression network analysis).

To evaluate the prognostic performance of the proposed method, CPR [7], WGCNA (weighted gene co-expression network analysis) [5], the approach of Wu & Stein [6], and our new approach were compared. We used the area under the curve (AUC) method provided by scikit-learn [19] to measure the prognostic accuracy and performed 10-fold cross validation. We used heuristics to select good hyper-parameters. Figure 2a shows the AUCs measured in several ways for different cancers. The figure reveals that, based on AUC, the prognostic accuracy is higher than that achieved by the other methods. 

**Figure 3:**
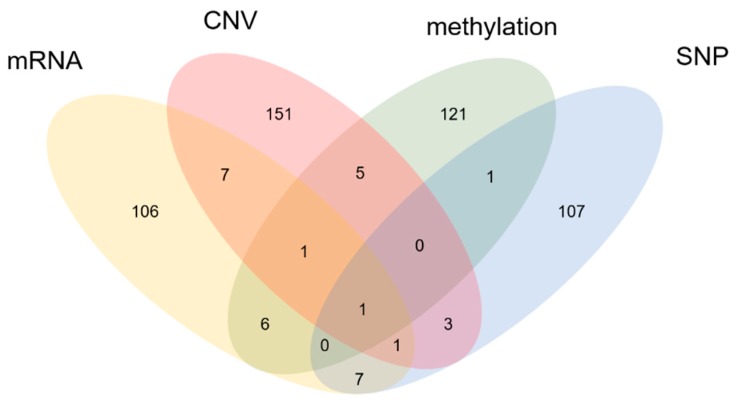
Number of biomarkers for each kind of omics data in PAAD. Venn diagram showing the biomarkers selected when applying our method to mRNA, CNV, DNA methylation, and SNP data.

To measure effects from using multi-omics data, we compared the AUCs using multi-omics data and each data type alone, for each cancer type. Figure 2b shows the AUCs of various data measured for several cancers. As the figure demonstrates performance in prognosis prediction is better when using multi-omics data than with single types of data, except in the case of STAD. For STAD, unlike the other cancer types, CNV had the greatest predictive power. This finding indicates that CNV could be a major factor in predicting the prognosis of patients with STAD. Moreover, multi-omics data shows much better performance in prognostic prediction for PAAD than other kinds of data. The reason for these results could be that the genes identified using each dataset are complementary to each other, as can be seen in Figure 3. This figure shows the genes that scored high for each omics dataset. Supplementary Table S3 shows the genes selected from each data set.

### 3.3. Oncogene Inclusion Test

**Figure 4:**
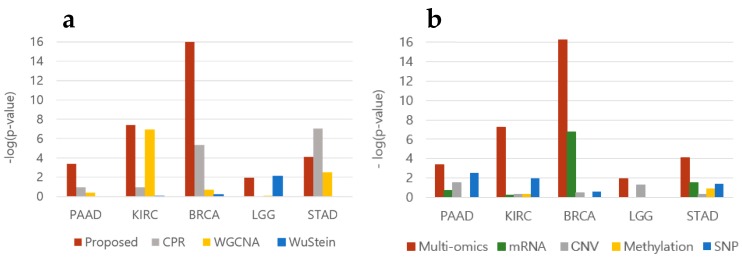
Significance of the oncogenes inclusion ratio for each (**a**) method and (**b**) omics dataset. Significance values of the oncogenes identified from intOGene were calculated using hypergeometric tests. Complete contingency tables are provided in Supplementary Table S4.

To evaluate the biomarkers we selected, we counted known oncogenes collected from intOGene [20,21], and calculated *p*-values using hypergeometric tests. Figure 4a shows that our *p*-values are lower than those obtained with other methods, with the exception of those for LGG and STAD. We also compared *p*-values when using multiple data sets. Figure 4b shows that *p*-values are lower when using multi-omics data, a result also supported by Figure 3. All contingency tables are provided in Supplementary Table S4. 

### 3.4. Functional Analysis

**Figure 5:**
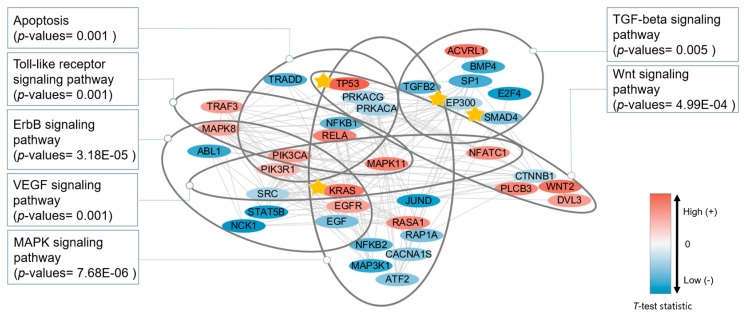
Network of prognostic genes for pancreatic cancer. Several enriched KEGG (Kyoto Encyclopedia of Genes and Genomes) pathways were selected and expressed. Genes with a star indicate known pancreatic cancer oncogenes.

To observe in detail the functions of the selected biomarkers, we performed functional annotation on biomarker targets using DAVID [22,23]. The detailed results from the functional annotation are provided in Supplementary Tables S5–S9. We selected the interesting KEGG (Kyoto Encyclopedia of Genes and Genomes) pathway [24] and visualized it simply using Cytoscape (Figure 5) [25]. Table 3 shows the genes in Figure 5. There are several KEGG pathways associated with the prognosis of pancreatic cancer, but we will first focus on the MAPK signaling pathway.

**Figure 6:**
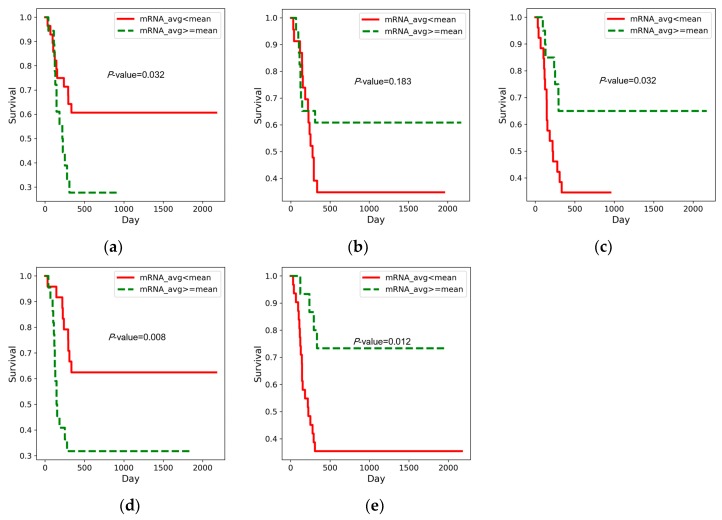
Kaplan–Meier graphs of (**a**) TGFB2, (**b**) NFKB1, (**c**) NFKB2, (**d**) EGFR, and (**e**) E2F4.

Pancreatic cancer is characterized by constitutive activation of the MAPK pathway [26]. Pancreatic cancer frequently involves mutations in KRAS [27], which contribute to the activation of MAPK, and active MAPK influences downstream genes that may play roles in malignant pancreatic cancer [26]. TGFB2, which initiates the MAPK signaling pathway, is known as a tumor suppressor, but in advanced disease, it appears to promote tumor progression [28]. TGFB2 is known to be involved in invasion by pancreatic cancer cells and correlates with patient survival after surgery [28,29,30,31]. We were able to observe that overexpression of TGFB2 is associated with the poor prognosis group in Figure 6a. 

**Figure 7:**
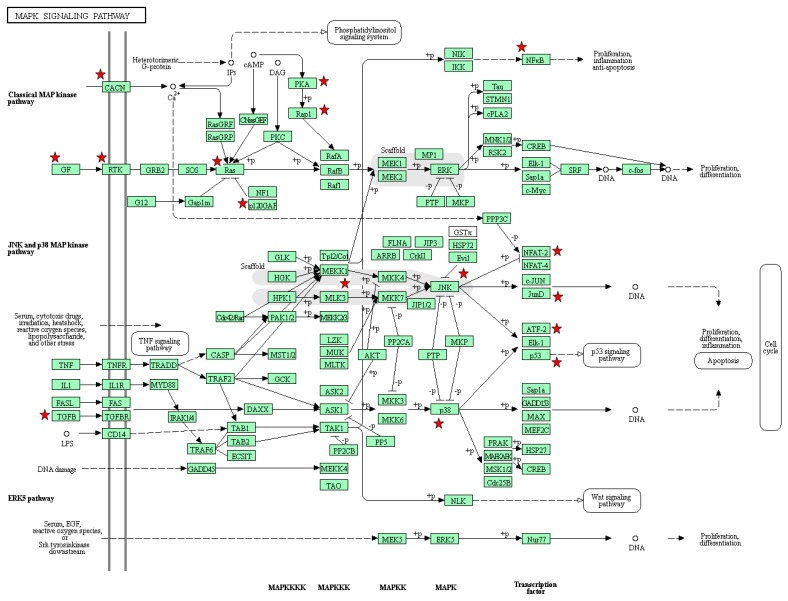
MAPK signaling pathway from KEGG database.

In the MAPK signaling pathway, one of the downstream genes of TGFB2 is MAP3K1. The effect of its mutation in multiple cancer types has recently been studied [32]. MAP3K1 has several downstream genes, as shown in Figure 7. One of them is NFKB (NFKB1 and NFKB2 in Figure 5). Constitutive activation of NFKB is observed in pancreatic ductal adenocarcinoma [33]. We found that the good prognosis group exhibits overexpression of NFKB1 and NFKB2 (Figure 6b,c, respectively). Another downstream gene, JunD is a subunit of the AP-1 transcription factor. This gene plays an essential role in pancreatic cancer cells [34]. We identified JunD by means of DNA methylation data (Table 2), and Figure 7 shows, using the KEGG pathway, that JunD, through DNA methylation, may affect apoptosis of cancer cells. TP53 is another downstream gene and a famous oncogene, which initiates the p53 signaling pathway.

The MAPK signaling pathway is associated with numerous signaling pathways, including the ErbB signaling pathway, which is also known to contribute to malignant tumor formation [35]. The overexpression of EGFR among our biomarkers is related to the prognosis of patients with pancreatic cancer [36,37]. We also observed that overexpression of EGFR is associated with the poor prognosis group (Figure 6d).

We were also able to observe the transforming growth factor-beta (TGF-beta) signaling pathway. This pathway does not overlap with the Toll-like, VEGF, Wnt, ErbB, and MAPK signaling pathways, which are, however, associated with each other (Figure 5). The TGF-beta signaling pathway regulates proliferation, apoptosis, and differentiation of cells, and induces resistance to the cytostatic activity of TGB-beta, while promoting the transformation and development of cancer into a malignant state [28,38]. In pancreatic cancer, this signaling pathway is associated with progression through angiogenesis and with decreased immunity [31,39,40]. E2F4 is one of the downstream genes of the TGF-beta signaling pathway and leads to the apoptosis of cancer cells. It has been determined that miR-17-5p promotes proliferation in pancreatic ductal adenocarcinoma cells by disrupting the RBL2/E2F4-associated gene repressing complexes [41]. We also observed that lower expression of E2F4 is associated with the poor survival group in Figure 6e.

## 4. Discussion

Many existing methods use the PageRank algorithm to identify disease genes or prognostic genes in biological networks [7]. However, owing to the nature of the PageRank algorithm, it was often found that too many hub genes, such as UBC, were identified, and non-hub genes were seldom identified, even though they were well-studied disease genes. We thought that this tendency could be moderated if the biological network could be specified. For this purpose, we first performed a *t*-test for each gene, for each type of omics data, and the FIs network was reconstructed with genes that showed a high absolute value of the *t*-value for at least one type of omics data. This process was effective for merging multiple-omics data. The findings showed that multi-omics data contributed to more accurate prediction of cancer prognoses, most likely because prognostic genes were complementary in multi-omics datasets. Moreover, Figure 5 and Table 2 show the abstract roles of genes in the context of a network. For example, DNA methylation by JunD may be related to cancer cell apoptosis.

We could generate a candidate FIs network using the *t*-test for each type of omics data, but this procedure is inefficient, because the PageRank algorithm is sensitive to the weights of the network. To determine the correct edge weights of the network, we used the GANs model to learn a correct FIs network. We applied the PageRank algorithm to the resulting candidate FIs network, and the top scoring genes showed higher prediction accuracy than did the preexisting methods. In addition, those genes were functionally meaningful.

DNNs have achieved breakthroughs in applications with large sample size. However, when facing high dimension, low sample size (HDLSS) data, DNNs suffer from overfitting [42]. In the case of the GANs model, overfitting is a main factor that prevents convergence, because overfitting refers to the lack of generality of the model, so the loss function of the GANs model that has overfitted generator cannot be converged to 0.5. To solve this problem, we replaced the original fully connected network of a generator with the reconstructed FIs network. Because the FIs network acts as a filter, the method is able to achieve sparse connection between network layers to prevent overfitting [43]. Also, since the initialization weight value is less than 1, the $w⊙w^{T}$ gradually approaches 0. This has the same effect as L2 regularization which is known as a technique to avoid overfitting. As a result, the GANs model with reconstructed FIs network can be a general model that converges even for small samples.

## 5. Conclusions

We proposed a novel machine learning-based method for more accurate prediction of cancer prognosis. We applied the proposed method to multi-omics data including CNV, mRNA, DNA methylation, and SNP data for five cancer types. The proposed method showed better prediction accuracy than existing methods, and we were able to identify numerous genes that are related to cancer development, as well as to reveal details about their roles in biological pathways. We also demonstrated that genes identified from each kind of omics data did not show much overlap, which led to improved accuracy in prediction when using multi-omics data.

## Figures and Tables

**Table 1:** Data information.

Cancer	#Gene in Multi-Omics	#Gene in mRNA	#Gene in CNV	#Gene in DNA Methylation	#Gene in SNP	#Good Sample	#Poor Sample	#Criteria for Label
PAAD	7371	11,404	11,637	11,509	7571	20	24	1 year
BRCA	7908	11,578	11,093	9216	10,354	91	63	5 years
KIRC	8801	11,569	11,643	11,508	8920	65	47	4 years
LGG	11,419	11,511	11,642	11,517	NA	66	59	3 years
STAD	5634	11,571	11,645	11,515	6836	29	16	1 year

Pancreatic adenocarcinoma (PAAD), breast invasive carcinoma (BRCA), kidney renal clear cell carcinoma (KIRC), brain lower grade glioma (LGG), and stomach adenocarcinoma (STAD). Copy number variants (CNV), single nucleotide polymorphism (SNP).

**Table 2:** Hyper parameter in GANs.

Epoch	Batch Size	Learning Rate	Optimizer
2	1	0.0002	Adam optimizer

**Table 3:** Genes selected from each form of omics data.

Data	Gene
mRNA	ABL1, E2F4, EGFR, NFKB1, NFKB2, PRKACA, RASA1, SRC, STAT5B, TGFB2, TRADD
CNV	DVL3, KRAS, MAPK8, NFATC1, PIK3CA, PLCB3, RELA
DNA methylation	ACVRL1, ATF2, BMP4, EGF, JUND, MAP3K1, MAPK11, NCK1, NFKB2, PIK3R1, PRKACG, RAP1A, SP1, TGFB2, TRAF3, WNT2
SNP	CACNA1S, CTNNB1, EP300, SMAD4, TP53

## Supplementary Materials

The following are available online at http://www.mdpi.com/2073-4425/9/10/478/s1, Table S1: Parameter in experiment, Table S2: Biomarkers selected for each cancer type, Table S3: Selected biomarker with only one dataset for PAAD, Table S4: Contingency tables, Table S5: Functional Annotation about biomarker in PAAD using DAVID, Table S6: Functional Annotation about biomarker in BRCA using DAVID, Table S7: Functional Annotation about biomarker in LGG using DAVID, Table S8: Functional Annotation about biomarker in STAD using DAVID, Table S9: Functional Annotation about biomarker in KIRC using DAVID.

## Availability

https://github.com/MinseonKim1107/Improved-method-for-prediction-of-cancer-prognosis-by-network-learning.
